# Innovation below the surface: development of a canine underwater search training device for submerged scent detection

**DOI:** 10.3389/fvets.2025.1653518

**Published:** 2026-01-06

**Authors:** Paul Bunker, Christina Brewster

**Affiliations:** Chiron K9, LLC, Somerset, TX, United States

**Keywords:** underwater detection, canine detection, oil spill, human remains detection, canine olfaction

## Abstract

The effective detection of targets in aquatic environments, particularly underwater, poses a significant challenge for environmental and conservation monitoring and response. Canines are used in a detection role to search for human cadavers, whale scat, invasive fish, and spilled oil. The use of canines for underwater detection is limited by our imagination, and in some cases, the ability to train the canines using the target samples. A significant challenge in training a canine for underwater detection, such as oil, is that the target (oil) cannot be placed in water environments, which could contaminate the water source. This paper describes the design, development, training protocols, and validation of a Canine Underwater Search Training Device (CUSTD), which is a novel, remotely operated system that facilitates the training of canines to detect underwater targets safely and, relatedly, without the need to place training materials directly in the water source. The device allows for trainers and handlers to deliver target odors from the front of a boat, therefore conditioning a detection canine to search from the front of a boat and to give a response (indication) when the target odor is detected. In October of 2024, a research study assessed the ability of canines to detect petroleum-based compounds underwater in controlled field conditions. Canines initially were trained to recognize the volatile organic compounds (VOCs) associated with crude oil underwater from a boat using the CUSTD. The innovation of the device provided the ability to expose the canine to target VOCs from the front of a boat without requiring oil to be placed within the water source. The methodology has wider potential applications, including environmental monitoring, oil spill response, search and recovery, and marine biology research. The CUSTD provides a versatile and ethical platform for advancing both scientific study and operational canine deployment in underwater detection disciplines.

## 1 Introduction

Canines have been trained and used for many years to locate submerged or sunken cadavers (1) and whale scat from the front of a boat (2). The use of canines in underwater detection is expanding, with trials demonstrating the ability of canines to detect fish (3) and the veliger of invasive mussels (4) from water samples. Canines have been trained to detect oil on shorelines following a spill or oil targets during controlled field trials in a range of terrestrial or shoreline environments (5) and have located oil underwater during operational deployments. Therefore, it was believed that canines would have the potential to detect submerged or sunken oil from a boat using the protocols developed for underwater cadaver detection.

Targets (or simulants) are placed in the water to train the canine to search and locate from a boat. However, this is not always possible, especially regarding oil and human body parts, due to environmental concerns and regulations. When the option is to hide targets in the water, they are placed in one location, and the canine either only receives one trial on the target or must repeat the training trial at the exact target location more than once. This limits training opportunities as the canine knows where the target is after the first trial, limiting the learning experience. It was therefore recognized that a specialized system was needed to train canines in underwater detection without introducing contaminants into the water source used for their conditioning.

Canines cannot detect targets through water itself. Instead, they detect gases, particulates, and dissolved molecules that rise to the surface. These light components of the target oil reach the surface of the water and may evaporate directly or disperse and then evaporate depending on environmental conditions (temperature, winds, wave action, etc.). The canines can detect targets underwater by detecting the scent on the water’s surface and/or in the air (6).

Detecting spilled oil underwater is significantly more complex than locating oil on the surface. Most conventional sensors do not work in deep water, and few are effective in depths less than 2 m (7). Oil detection canines (ODCs) have proven to be highly effective in support of shoreline oil spill response surveys (5, 8–10). ODCs have a very low detection threshold and have successfully located weathered oil targets on land up to 5 m below the surface (10). In addition, they are highly selective and can be trained to find specific oil in multiple oil deposits (11). The speed and accuracy of ODCs in locating oil deposits on shorelines is a significant improvement over other techniques and may similarly help to detect oil in non-traditional settings where existing methods are challenged or ineffective.

The continuously expanding capabilities of ODCs and the potential to improve spill response outcomes were recognized recently by the US Inter-agency Coordinating Committee on Oil Pollution Research (Arduino Nano BLE on a SenseTM board). The Plan identified two priority research needs that merit further examination. Specifically, the Plan noted the need for:

- Identifying and developing new methods of detecting, monitoring, containing, and recovering sunken or submerged oil.
- The expansion of oil detection canines’ (ODCs) capabilities to support shoreline spill response surveys and operations.

In 2016, four ODC teams were deployed to support a Shoreline Cleanup Assessment Technique (SCAT) program during an oil spill response on the North Saskatchewan River (12). During several surveys, the handlers observed that canines would wade into the river and exhibit a change of behavior—a specific behavior type indicating that the canine smells the target odor. On these occasions, further investigation of the area included disturbing the river sediment that released oil to form a sheen on the water’s surface.

In addition, during a series of beach tarball surveys on the Texas Gulf Coast, the canine was observed exhibiting a change of behavior, then going into the sea and following tar balls to the shore as they washed up.

Based on these observations, the Bureau of Safety and Environmental Enforcement (BSEE) funded the US Naval Research Laboratory (USNRL) in 2021 to investigate the detection of oil underwater, and part of the project included a study with ODCs. A research trial developed protocols supported by a specifically developed underwater training odor delivery device, which resulted in the ability to train ODCs without any risk of releasing oil into the environment (13). Although the device proved effective, it had limitations for training protocols, therefore a second device was developed (14).

Combining the training protocols and utilization of the two underwater training devices previously developed resulted in the capability to train canines to detect oil underwater. The positive results in training led to a large-scale investigation of submerged and sunken oil using ODCs at the International Institute for Sustainable Development (IISD) Experimental Lakes Area (ELA) in western Ontario, Canada, in September 2024. The focus of this paper is to describe the development of the methodology and the field validation of a novel canine training device specifically designed to condition detection dogs to identify submerged and sunken oil in aquatic environments.

## 2 Materials and equipment

Bunker et al. (13) describe the development and testing of an underwater odor delivery device utilized in the training of oil detection canines (ODCs) to detect submerged and sunken oil without risk of environmental contamination. Field observations had suggested that dogs could identify oil sequestered in sediments underwater, but formal training was limited by the inability to place oil samples in aquatic systems safely. The 2022 study introduced the initial iteration of an Underwater Detection Device and associated protocols, allowing canines to be conditioned to detect oil plumes from shorelines and while searching from the front of boats. Informal laboratory and field trials demonstrated that dogs could reliably locate crude oil odors in water, and headspace analysis confirmed that water alters the volatile profile of oil, but that sufficient odor is available for detection (13). However, the first underwater detection device had some limitations. The device takes time to be installed into a water source as the pipe transporting the headspace odor of the oil must be weighed and laid by boat within the lake or river. This also means that once deployed, the device has limited training value before it must be moved to a different location, as the canine may quickly learn the area where the odor will be encountered rather than searching for the odor only. Additionally, the device produces bubbles of odor, which, although controlled, may provide an auditory and visual indication that the target odor is present. This paper describes a second device, the Canine Underwater Search Training Device, which was developed to mitigate the limitations of the underwater detection device as a follow-on step in the training protocols.

**Figure 1:**
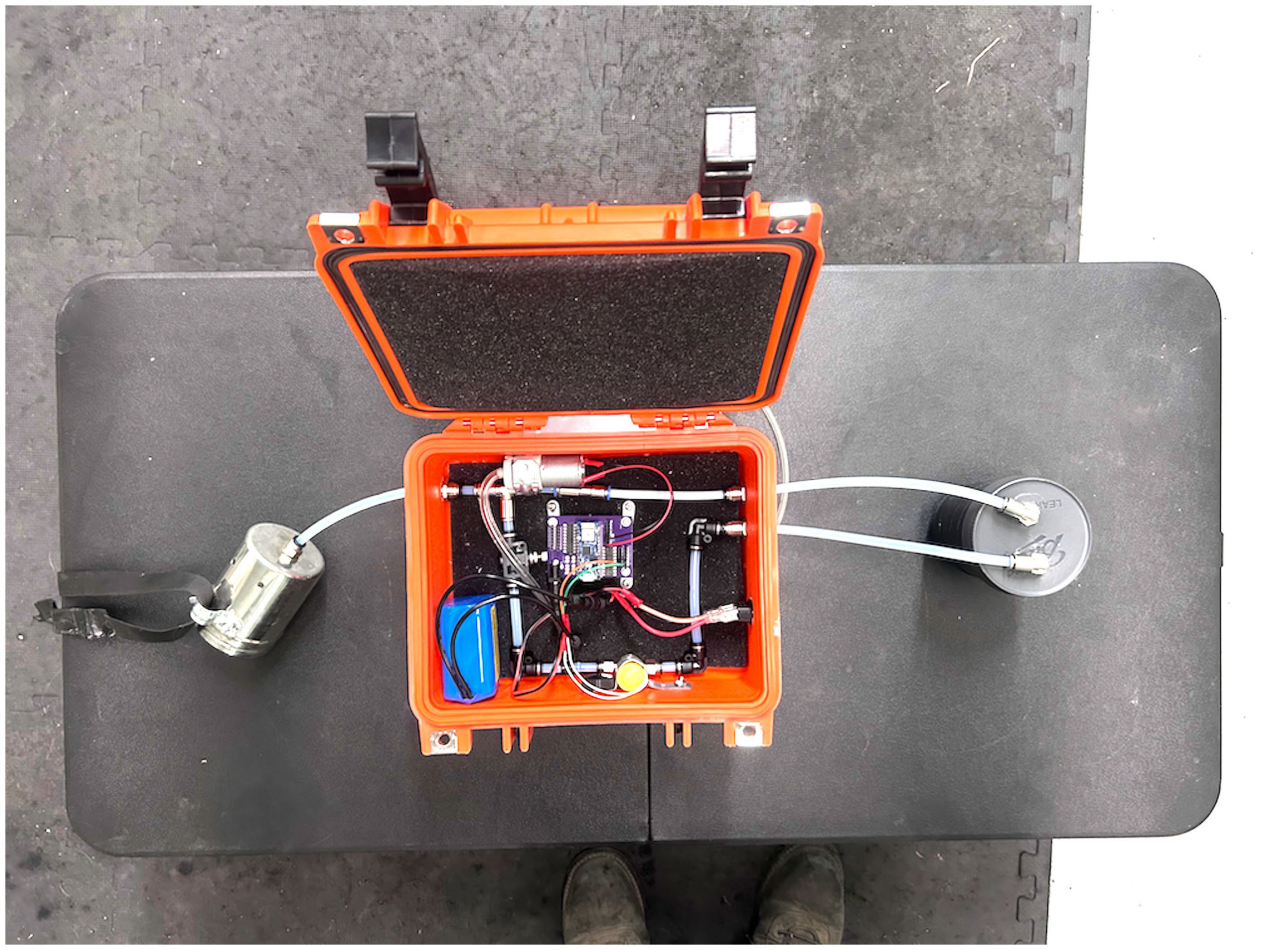
Canine underwater search training device.

**Figure 2:**
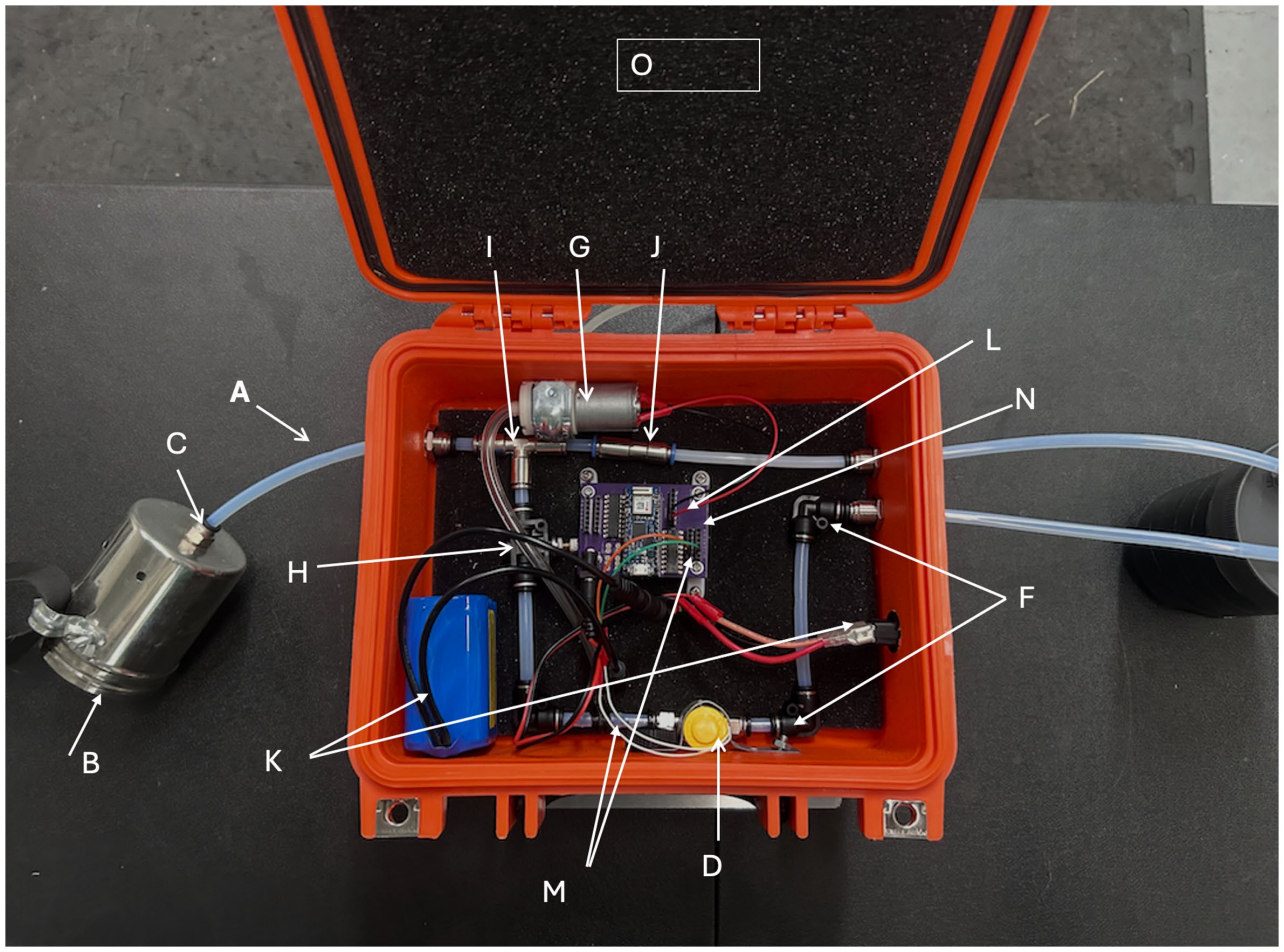
Schematic of the canine underwater search training device.

The Canine Underwater Search Training Device (Figures 1, 2) was designed to train detection canines to search from the front of a boat for targets on or under a water source. The device can be used with any target odor on which a canine may be required to search from a boat. Examples are, but are not limited to, cadavers, whale scat, fish, and oil. Currently, canines are trained on target sources by placing target samples in or under the water. This limits the training session to one trial or to repeating the same target odor at the exact location multiple times. The second device allows for repeated presentations of the target odor and is remotely controlled by the canine trainer. The device only emits target odors when the trainer operates the device, providing control over training trials, which can be repeated many times within a training session. The odor delivery port is located at the front of the boat, allowing the dog to sniff over the boat’s bow and search for target odors emanating from the water.

Canine underwater search training device (Figures 1, 2).

### 2.1 Parts

**Figure 3:**
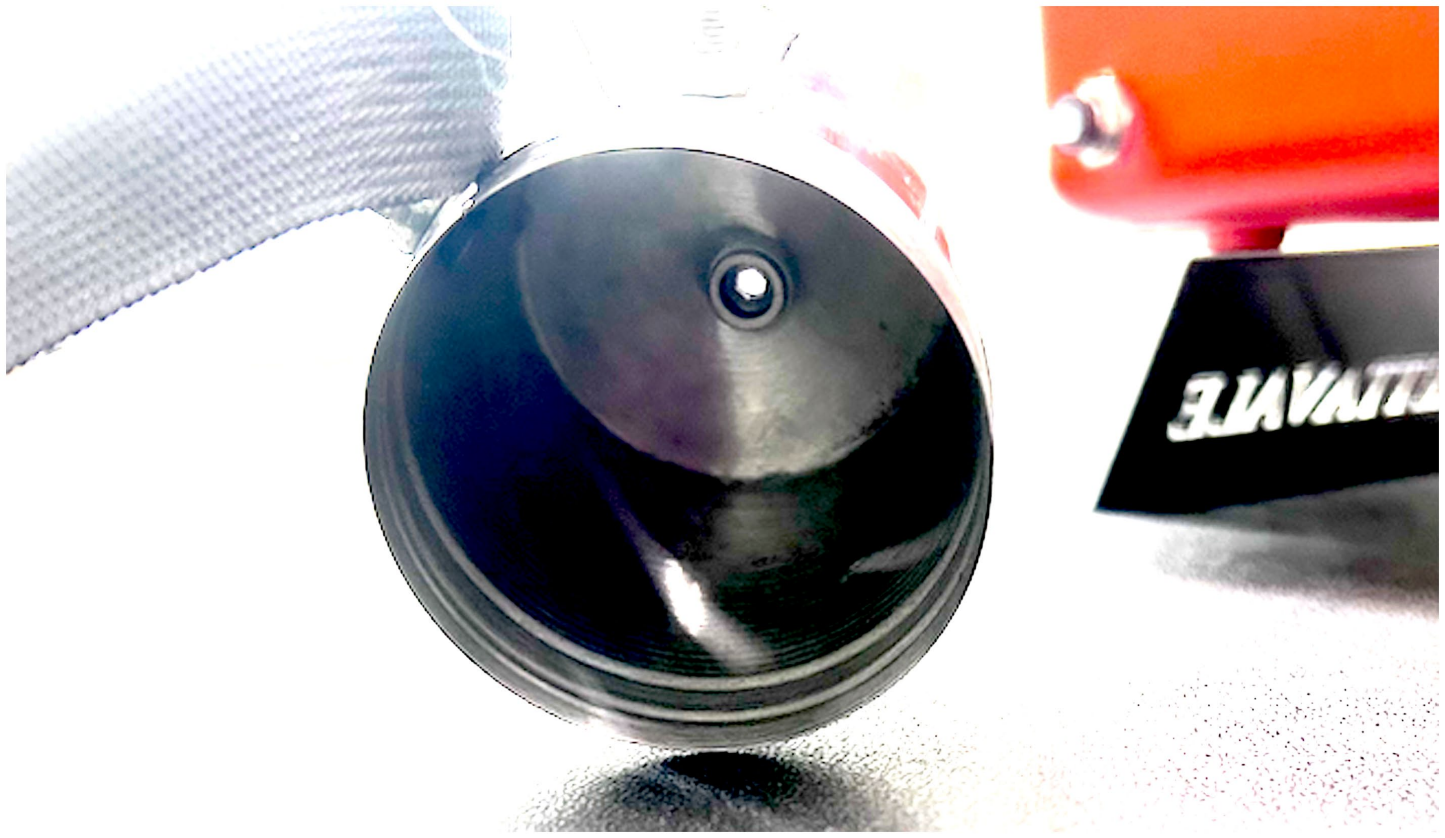
Inside the spice shaker (Part #2) odor delivery port.

**Figure 4:**
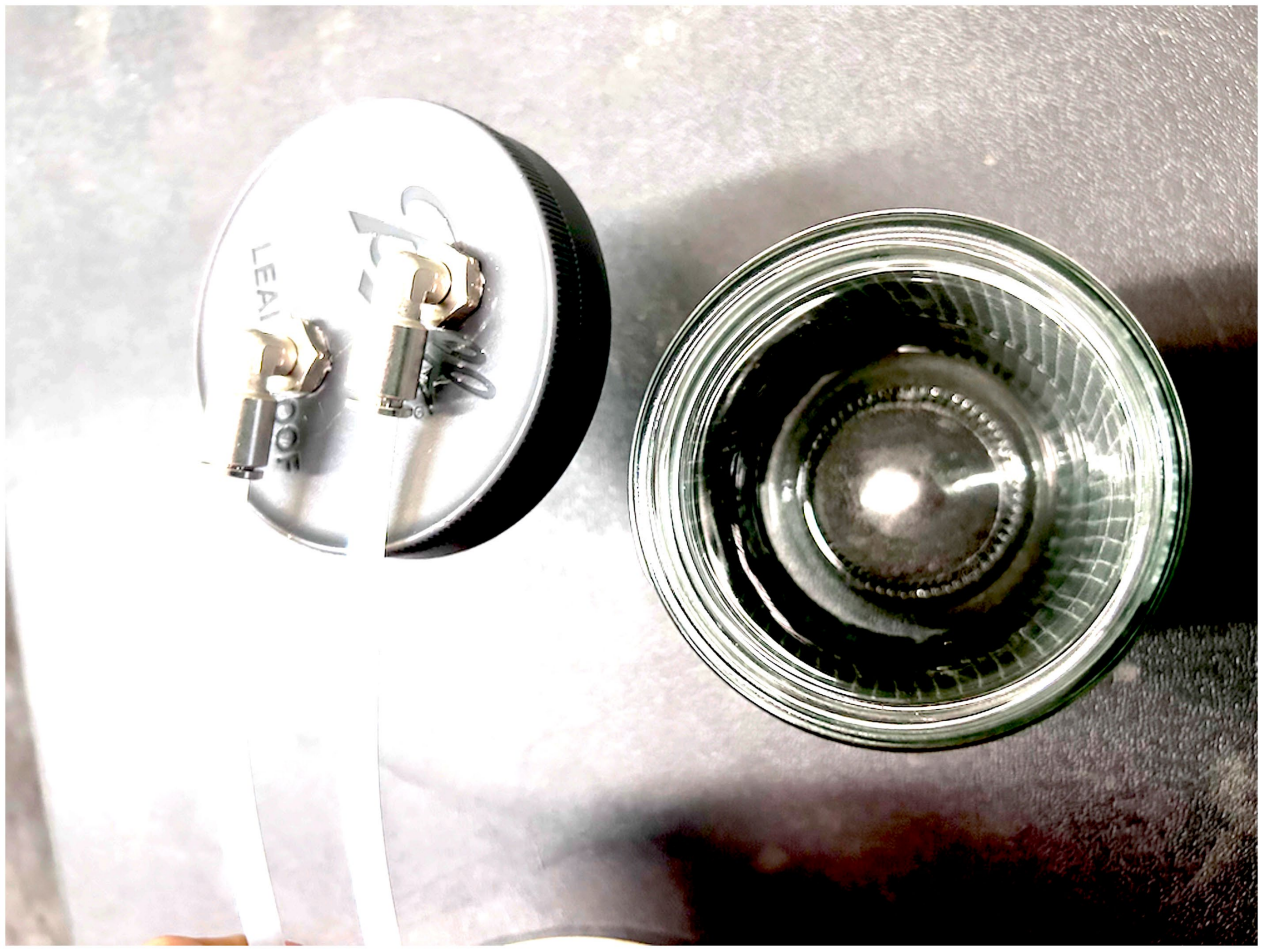
Part #5 Glass Mason jar, which holds the target sample.

- Polytetrafluoroethylene (PTFE) 3 m of tubing 4 mm internal diameter, 6 mm outer diameter (OD).
- Odor port is made from a stainless-steel spice shaker (Figure 3).
- Odor port to PTFE pneumatic stainless-steel push connector 6 mm OD.
- Clippard EV-2-12-H “normally closed” Two-way Electronic Valve with push connectors 6 mm OD.
- Glass Mason jar with stainless-steel lid (Figure 4).
- Stainless-steel push-connect elbow connectors - 6 mm OD.
- Jadeshay 12 V electronic air pump.
- Airflow control valve 6 mm OD.
- Union Tee Push to connect fittings stainless steel quick connect fitting 6 mm OD.
- Stainless steel check valve 6 mm OD push connector
- Power source and switch.
- Electric power wires for the air pump.
- Electric power wires for the two-way electronic valve.
- Arduino Nano, IOT Bluetooth-enabled power control board.
- Waterproof case.

The odor port (B) (Figure 3) is attached to the front of a boat to train and deploy canines to detect targets on or underwater. Tubing (A) from the port is positioned directly into the boat or along the side and attached to a container (O), which protects the device from water and damage during transportation.

The device is switched on to provide power to the Arduino Nano 33 IoT Bluetooth-enabled on a custom printed Power Control Board (15) that serves as the operating system. The control board provides power to the air pump (G) via power wires (L) and to the two-way electronic valve (D) via power wires (M) and operates the device’s function. When the device is switched on by using the power button (K), the air pump (G) starts to push ambient air through the system’s tubing. The intake tube for the air pump is fed through the container wall to the outside and collects ambient air to push through the system. Ambient air is pushed out of the pneumatic push connector (C) into the odor port (B). An airflow control valve (H) reduces the air pressure in the clean air system so that the air follows the path of least resistance into the target sample jar when the device is switched to present the target air. The air then leaves the odor port and enters the atmosphere to dissipate.

The device is operated by a remote switch such as a mobile phone, tablet app, or Bluetooth-enabled button. When the Bluetooth-enabled button is functioning, the two-way valve (D) is opened, and air containing the target odor is pushed from the vial (E) to join the ambient air and moves through the tubing (A) and out the odor port (B) via the pneumatic push connector (C). When the Bluetooth-enabled button is next pressed, the two-way valve (D) is closed, and air containing the target odor is stopped. Clean ambient air is pushed through the tubing (A) and out the odor port (B) via the pneumatic push connector (C) to clear the system.

The odor port (B) is attached to the front of a boat for the field investigation. The tube (A) leading from the port is run along the side of the boat, and at a convenient point over the side and into the boat to a box (O) that protects the device from water and being knocked.

The air intake tube from the air pump is fed through the box and outside the boat to sample ambient air from the local environment. When the power switch (K) is operated, the system starts to pump ambient air via the pump (G) through the system. The system also boots up the Arduino (N) and activates the Bluetooth connection so that the device can connect to the operating app. The system utilizes an “Arduino Manager” (16) application on an iPhone or iPad. Arduino Manager connects to the device via Bluetooth, allowing the odor valve to open and close by pressing an in-app button. The app supports simple coding and custom development of widgets. The code sketch running on the Arduino board allows the Arduino Manager app to send or receive data from the board and control the device.

Because the two-way electronic valve (D) is closed on startup, only ambient air is emitted via the odor port on the front of the boat. The ambient air is constantly supplied once the power switch is operated.

The sample glass Mason jar (E) contains the target material upon which the canine is trained (Figure 4). When the pneumatic push connector (D) is opened by the Bluetooth-enabled power control board (N), air from the air pump (G) is pushed into the jar; this forces the odor headspace of the target material out of the jar through the tube via Check Valve (J) and to the odor port (B). The sample vial can be any size of container to suit the requirements of the target training sample.

When the Arduino Manager in-app button is set to “on,” the two-way electronic valve opens and ambient air flows into the Mason jar containing the target odor. When the in-app button is set to off, the two-way electronic valve closes, preventing air from flowing into the Mason jar containing the target odor, meaning only ambient air is being pushed through the system. Once the air containing the target VOCs exits the port (B), it disseminates into the ambient air.

The flow of ambient air cleans the Teflon piping of any residual target odors and allows the system to be operated again (16). The cleaning process is timed at a minimum of 45 s to ensure the system contains no residual target odor. However, the flow rate can be calculated using the Continuity Equation for the steady-state flow equation, and for this device setup, it is calculated as 0.7 s, but factors such as the type of target odor used would have an influence on that time. Oil being very persistent resulted in extending the time to ensure there is no residual odor.

The result is that the target odor can be delivered to the odor port whenever the trainer or assistant desires the canine to encounter the target odor. The duration of the presentation is also fully controlled by the trainer or assistant. Additionally, an assessor can operate the system without the handler knowing when the target odor is presented for controlled testing of the canine team.

## 3 Methods

A 6-year-old English Springer Spaniel (“Poppy”) was utilized for the project. Poppy is an experienced and certified Oil Detection Canine that has supported multiple research oil detection projects and spill response deployments. Poppy is credited with thousands of confirmed field finds that include weathered hydrocarbons. Poppy has demonstrated a zero false-positive response rate in double-blind research trials (17–19) previously and did not give any false-positive alerts throughout the training phase.

Poppy is a generalist ODC, which means she is trained to detect and alert to hydrocarbon samples, including weathered and unweathered oils. The other type of ODC is a Specific Oil Detection Canine (SODC), and these are trained to detect and alert to unweathered samples of hydrocarbon and ignore weathered samples (11, 20). Two oil types were selected for training using the Canine Underwater Search Training Device (Table 1).

**Table 1:** Characteristics of the two oil types used in the canine training program.

TEST OIL	Description and density
Bunker C	Bunker C (a.k.a. number 6 fuel oil, heavy fuel oil) is a residual fuel oil used for propulsion on some marine cargo vessels. It has a tar-like consistency and must be heated to flow. Density: 0.9912 g/mL @ 10 °C
Dilbit/Heavy Crude Mix	This oil was a mix of conventional crude oils and diluted bitumen oils commonly transported in pipelines in the Great Lakes area. The constituent oils included Albian Heavy Synthetic, Conventional Heavy, Cold Lake Blend, and Light Sour Blend. All constituent oils were artificially weathered in a wind tunnel. Density: 0.9995 g/mL @ 10 °C

The two oil types, Bunker C and Dilbit (diluted bitumen), were chosen for training as these are two of the products selected for future trials at the Experimental Lakes Area (ELA) in Canada. Both products had been previously used in research trials (17) and were available for training and testing.

A systematic training protocol ensured that Poppy recognized the two targets before introducing the Canine Underwater Search Training Device.

### 3.1 Step 1: Oil in jars

For the first step, 100 mL of sample oil was mixed with 50 g of sand and placed in a glass Mason jar. Two other Mason jars were prepared with sand, but no oil target. The jars were placed side-by-side in a linear configuration, and Poppy was allowed to investigate them. If she was alerted to the jar containing the oil sample, it was marked (use of a clicker sound), and she was rewarded. Poppy had no problems locating and alerting to the Dilbit and Bunker C.

### 3.2 Step 2: Oil submerged in jars

After Poppy detected and alerted the two target samples within the jar lineup, distilled water was added to the jars to submerge the products. The process was repeated, and Poppy had no issues detecting and responding to Dilbit and Bunker C underwater.

### 3.3 Step 3: Oil submerged in buckets

Once Poppy consistently detected and alerted to the two submerged samples within the jars, the process was replicated using 5-gallon buckets. Four 5-gallon buckets were cleaned with bleach water and air-dried in the sun to remove contamination. Approximately 1 kg of sand and 250 mL of each oil sample were mixed and placed in two buckets. Three gallons of distilled water were then added to each of the buckets. The remaining two buckets had three gallons of water added to the sand. Lids were placed on the buckets, and the products were left to settle for 24 h to ensure that no sheen formed on the surface.

Three holes were drilled into the bucket’s plastic lids and placed on the buckets so that odor could escape, but Poppy and the trainer could not see the contents. This ensured that the training sessions were blind and uninfluenced by the trainer. The buckets were placed five meters apart in a row, and Poppy was released to investigate each bucket. Upon the correct selection of a bucket containing the target oil sample, a clicker sound communicated the correct choice, and Poppy was rewarded. Poppy detected and alerted to the Dilbit and Bunker C without issue.

### 3.4 Step 4: Canine underwater search training device

Once Poppy demonstrated consistent detection capability for the samples in buckets, the training transitioned to the Canine Underwater Search Training Device.

**Figure 5:**
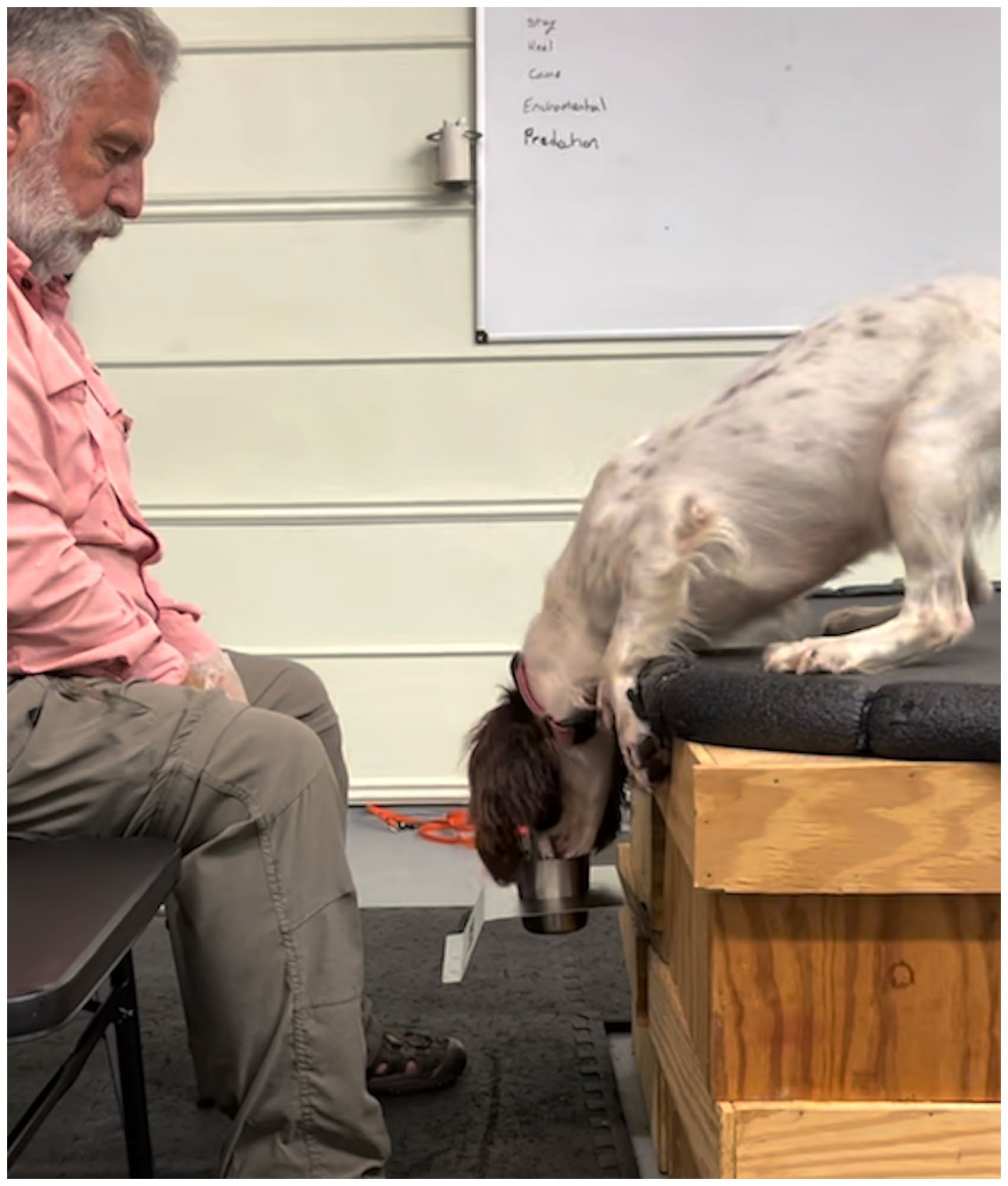
Poppy being introduced to the device in a training lab.

The first step to introducing the device was completed inside a training facility. A rubber-coated platform used on the boat’s bow was placed on blocks at the same height as the boat would be above the water’s surface. The odor port was suspended below the platform in front of where the canine’s head would be during a search. The canine was conditioned (trained) to lie on the platform with its nose near the odor port (Figure 5). In the initial step, no searching behavior was required to hold the correct position, and the odor port was not connected to the CUSTD.

### 3.5 Step 5: Operating the canine underwater search training device

The next step is to attach the CUSTD to the odor port via the 3-m Teflon pipe. The device is switched on, so clean air is being blown through the device, and the canine becomes accustomed to the air flowing, but remains neutral as no target is present currently.

The next step involved introducing the odor being transmitted through the device and then shaping a response (alert) behavior for Poppy when she detects the target. At this point, the Dilbit oil sample was added to the device. As Poppy uses a “sit” response when detecting oil, it was decided to use the same behavior on the boat. When the device was operated to permit odor to be present, and Poppy was observed to demonstrate changes of behavior (CoB) such as excitement, tail wagging, and whining, she was lured into a sit position and rewarded. This was repeated three times before the luring was stopped, and Poppy was rewarded when she was given a sit alert without being lured.

This step was repeated until Poppy would provide a sit response ten times in succession when the odor was presented.

### 3.6 Step 6: Introducing the canine underwater search training device on a boat

**Figure 6:**
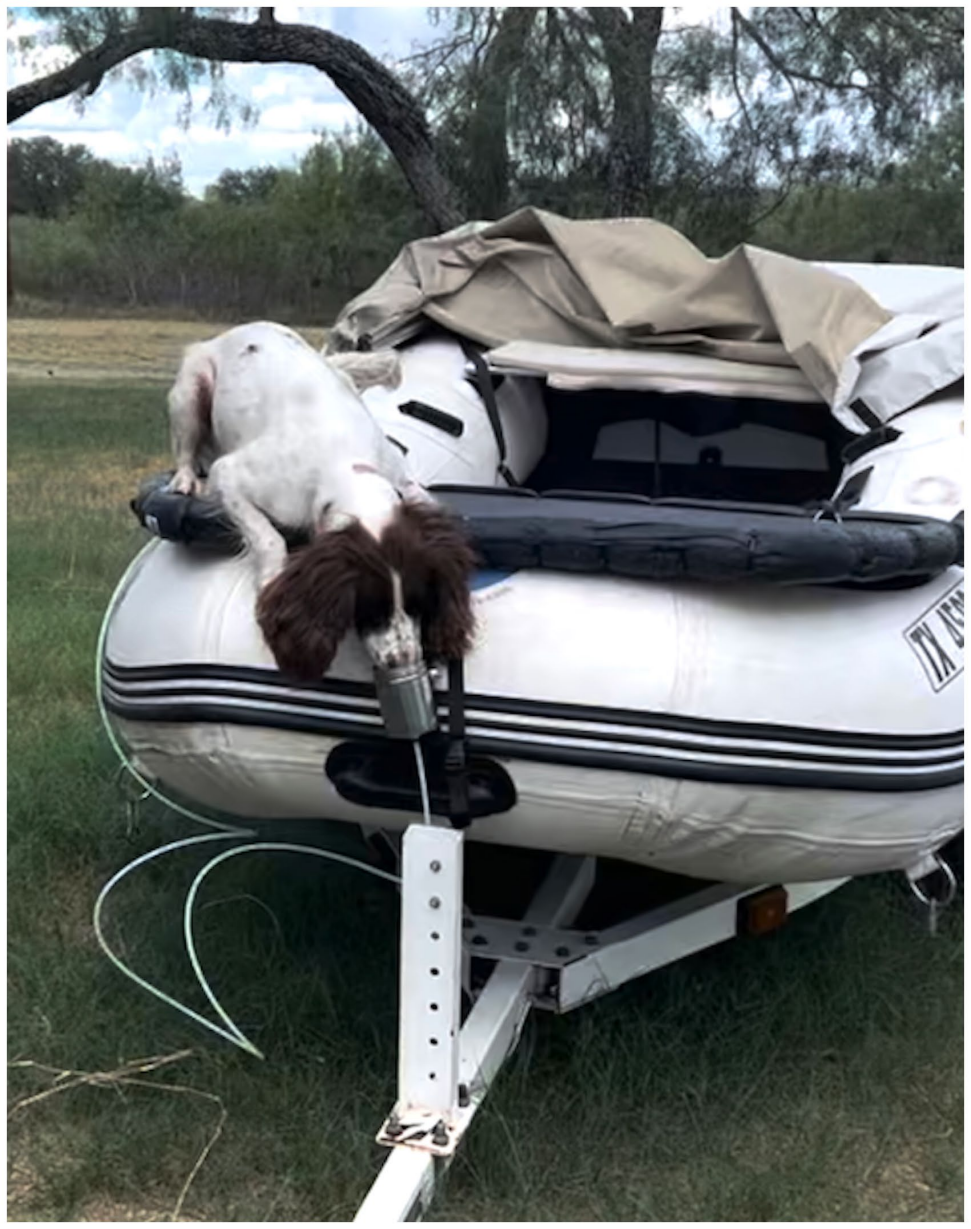
Poppy being trained in a boat away from the water.

The same rubber-coated platform was attached to the bow of a boat. The odor port suspended below the platform on the boat’s bow. Poppy was introduced to the device as before, but now outside and on the boat (Figure 6), and Step 5 was repeated.

**Figure 7:**
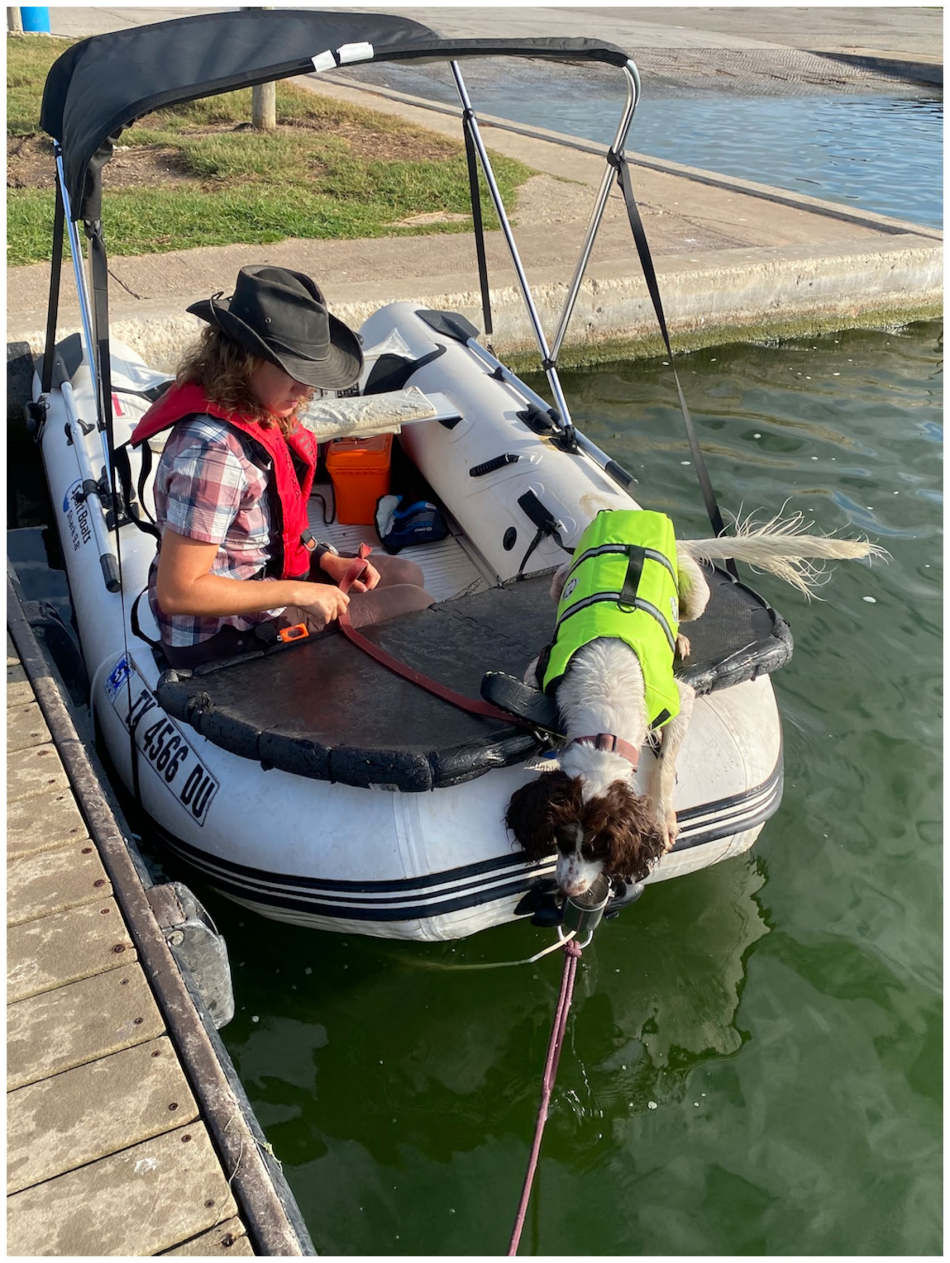
Poppy being trained with the device at the dockside.

Once Poppy had achieved 10 responses to the target odor, the boat was transported to a local lake, and Step 5 was repeated while the boat was in the water but secured to the dock (Figure 7).

The boat was then taken out to the lake, and the process was repeated.

After Poppy had responded 10 times in succession, Step 5 was repeated with the Bunker C oil sample.

### 3.7 Step 7

The boat was piloted onto the lake with the Canine Underwater Search Training Device odor port fitted to the bow. The device was switched on to deliver ambient air to the port. Poppy was given the search cue of “find it” and allowed to take a position on the front of the boat with her head over the edge to enable detection of any target odors. The boat was driven for 2 min before the odors were active within the device via the Arduino app. Upon sniffing the target odor, Poppy gave a sit response on the boat’s platform and was reinforced with a toy reward. This process was repeated 10 times.

### 3.8 Step 8: Underwater odor introduction

The target oil was placed in the Mason jar, and the protocols for searching from the boat’s bow and response were established. A simulated underwater target odor headspace is introduced at this stage. 100 mL of oil was mixed with 50 g of sand and placed in a Mason jar. Then distilled water was added to the jar. Now, the headspace generated by the device at the port is that of sequestered oil in sediment, which has traveled through water, giving a realistic representation of target odors. The same protocol as in Step 7 was repeated, with the canine being required to provide ten responses to the delivery of targeted odors.

## 4 Results

Upon completion of the field tests, the Canine Underwater Search Training Device was functioning as intended and delivered odor to the odor port when the app was activated and then stopped when the app was deactivated. The canine detected the target odor and exhibited a trained response.

**Figure 8:**
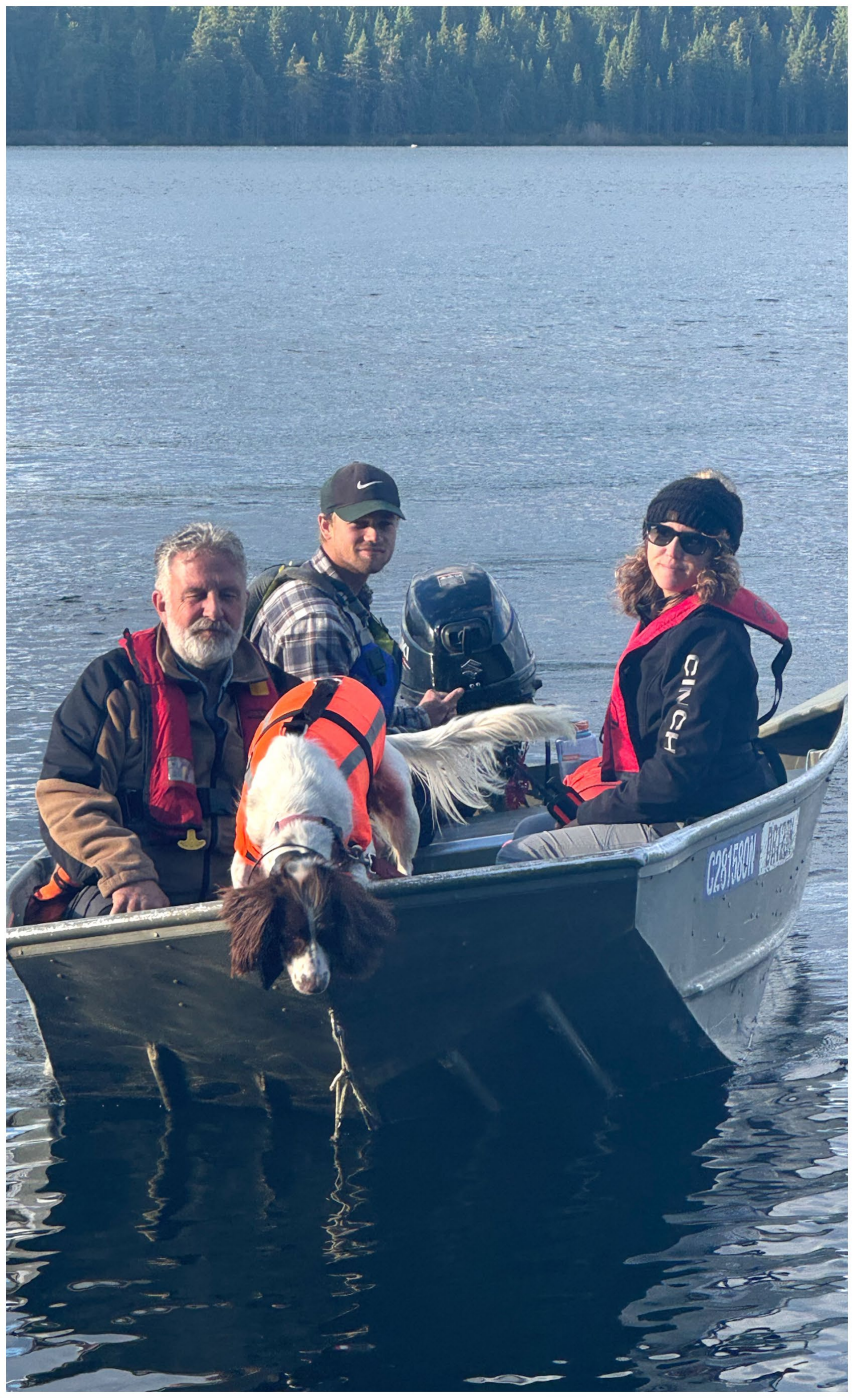
Field trials at the ELA in Ontario, Canada.

The large-scale field trials were conducted at the Experimental Lakes Area (ELA) in western Ontario, Canada. The ELA has specific provincial and federal legislation that permits researchers to conduct experimental studies that include the deposit or placement of deleterious substances, such as oil. The ability to spill oil in a controlled manner in a natural environment is a unique advantage of the ELA research station. A research trial was conducted using oil samples placed at different depths on the bed of a lake (Figures 7, 8), and the results are detailed in Bunker et al. (21).

In this example, the target is 250 mL of Bunker C oil 3 m on the bed of the lake. The canine provided two alerts for oil #15 and #16 and one alert for oil #17. It is assumed that alerts #094 and #097 are the detectable extent of the odor plume coming from the lake’s surface. Alerts #093 and #095 are the locations of the odor reaching the surface due to underwater currents. There were no other recorded alerts by the canine during this trial (Figure 10).

**Figure 9:**
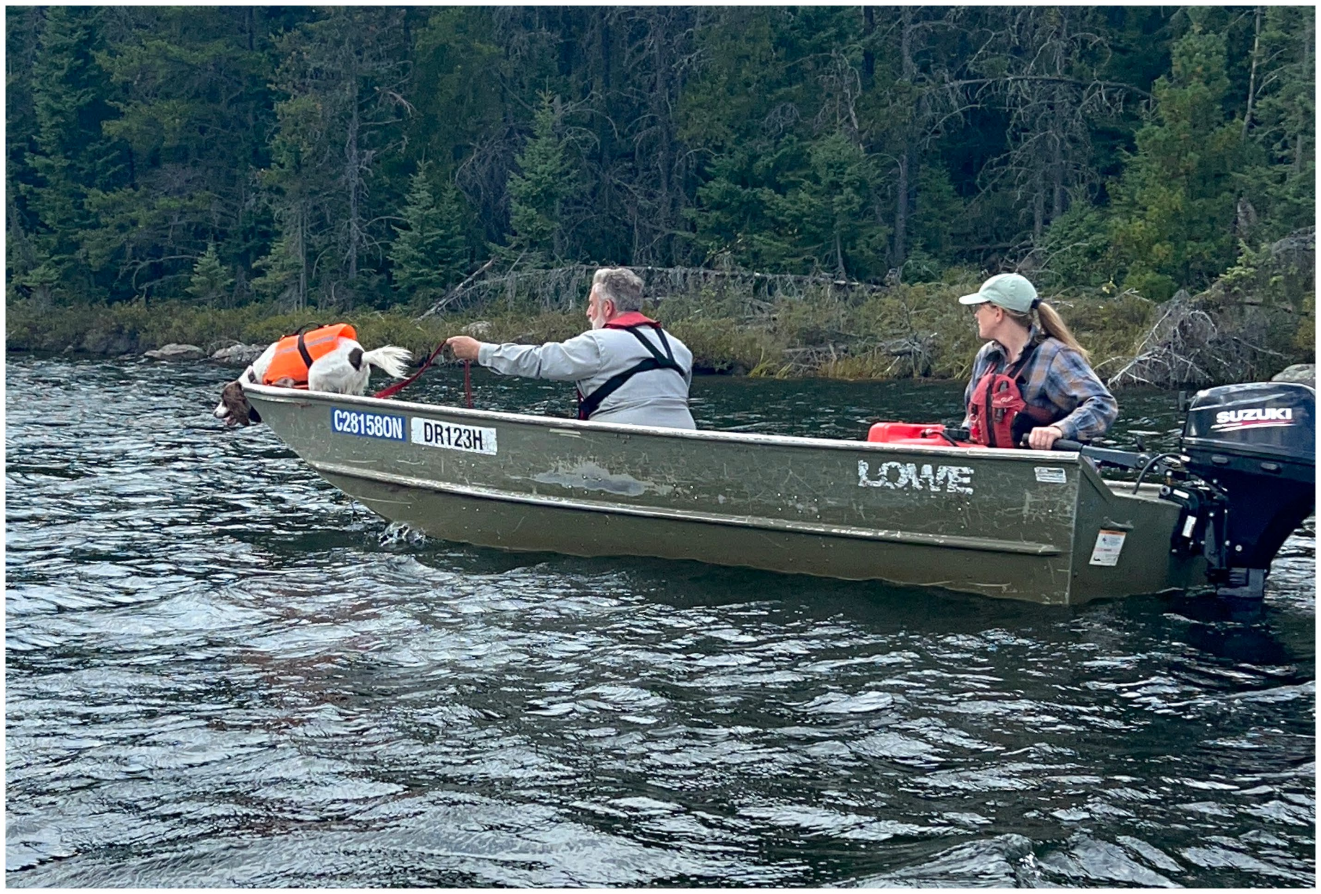
Poppy searching for submerged oil from a boat at the ELA.

**Figure 10:**
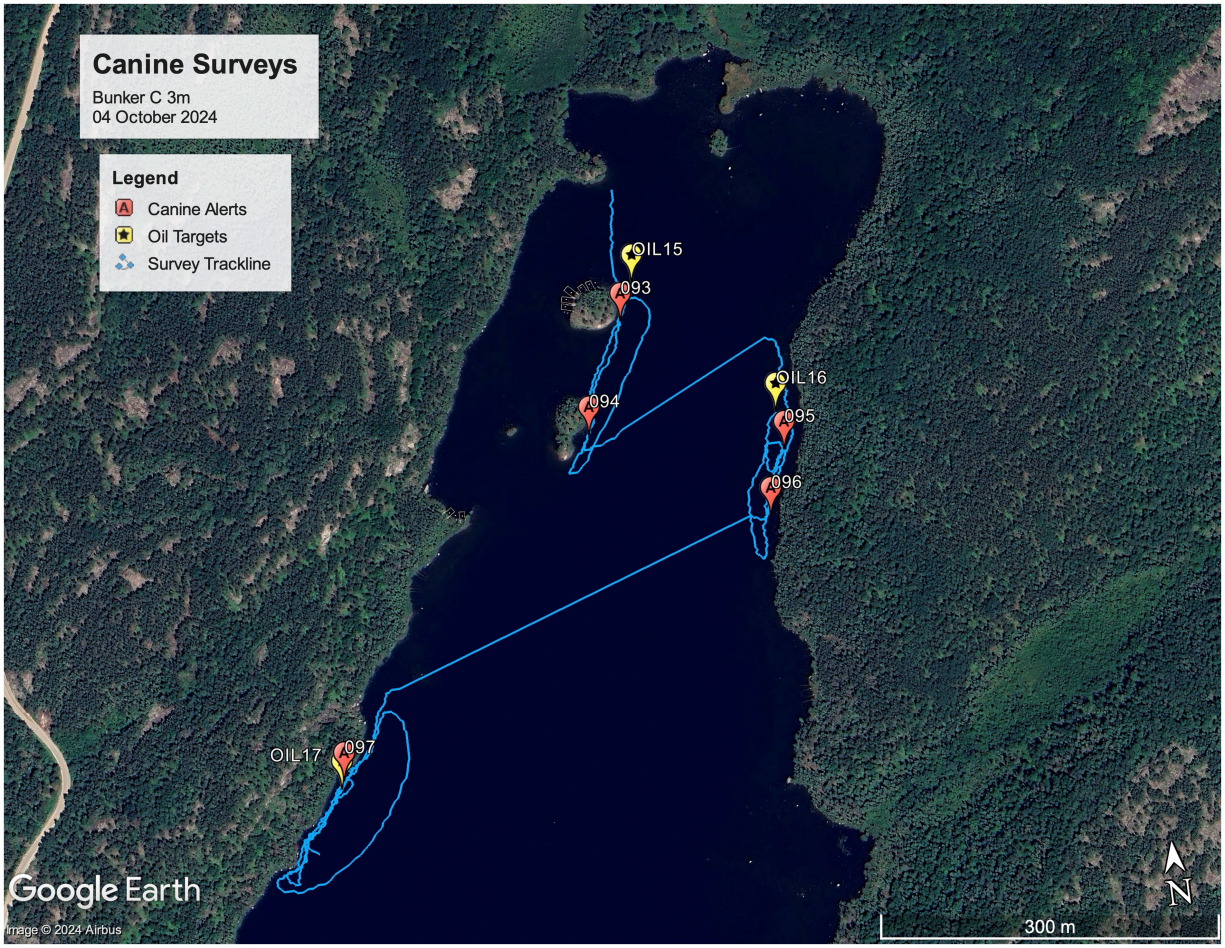
Example of oil locations and canine alerts during the ELA research trial.

## 5 Discussion

The Canine Underwater Search Training Device functioned as intended and provides a versatile method of delivering target odor remotely. The device provides a tool to train a canine in underwater target detection without contaminating the environment by placing targets directly in the water source.

Additionally, because the device is a standalone system for training, the canine can be trained using the device within a training room or on a boat that is not in the water. This allows training to be conducted in a controlled environment and to continue to the water once the canine understands the protocols of searching from a boat.

This trial was limited to only one canine for the training and field deployment due to the restriction that only weathered oil types could be used in the ELA lakes, and Poppy is the only canine that is trained to detect weathered oils. Previous field trials on land with up to four canines and multiple searches run over shallow and deep targets (8, 10) present quantitative data that objectively demonstrate successful replication. Other controlled studies with multiple canines (20) provide data on successful discrimination (18), and in a 2024–2025 multi-month beach study, an ODC recorded 825 indications (alerts) of targets, which were all verified and zero false positives.

Although data on odor concentrations from the device in the field would be interesting, unfortunately, this is not a practical option due to instrumentation limitations. Rigorously designed laboratory and field studies have documented that canines can detect odors at concentrations below the capability of existing laboratory instruments (22). The considerable variation in VOCs being emitted by the device, based on the target sample used, if placed underwater in the device, and ambient temperature, means there would be variation in the odor concentrations.

Further research into the device could benefit Human Remains (cadaver) Detection Dogs as well as Specific Oil Detection Canines. The device could also support the expansion of detection canine utilization for underwater targets such as invasive or endangered aquatic species and contamination response. Additionally, deploying the device in diverse climatic and environmental conditions would support its use in other projects.

## Data Availability

The raw data supporting the conclusions of this article will be made available by the authors, without undue reservation.
