# Correction: Innovation below the surface: development of a canine underwater search training device for submerged scent detection

**DOI:** 10.3389/fvets.2026.1798479

**Published:** 2026-02-16

**Authors:** Frontiers Production Office

**Affiliations:** Frontiers Media SA, Lausanne, Switzerland

**Keywords:** underwater detection, canine detection, oil spill, human remains detection, canine olfaction

A correction has been made to **Section 2.1** Parts, paragraphs 1–9.

In the published article, the parts of the canine underwater search training device visible in Figure 2 were identified with letters, as per the correct list in **Section 2.1**. Numbers 1–15, however, were used in paragraphs 1–9 of the same section, causing confusion.

The corrected paragraph of **Section 2.1** appears below:

“The odor port (B) (Figure 3) is attached to the front of a boat to train and deploy canines to detect targets on or underwater. Tubing (A) from the port is positioned directly into the boat or along the side and attached to a container (O), which protects the device from water and damage during transportation.

The device is switched on to provide power to the Arduino Nano 33 IoT Bluetooth-enabled on a custom printed Power Control Board (15) that serves as the operating system. The control board provides power to the air pump (G) via power wires (L) and to the two-way electronic valve (D) via power wires (M) and operates the device's function. When the device is switched on by using the power button (K), the air pump (G) starts to push ambient air through the system's tubing. The intake tube for the air pump is fed through the container wall to the outside and collects ambient air to push through the system. Ambient air is pushed out of the pneumatic push connector (C) into the odor port (B). An airflow control valve (H) reduces the air pressure in the clean air system so that the air follows the path of least resistance into the target sample jar when the device is switched to present the target air. The air then leaves the odor port and enters the atmosphere to dissipate.

The device is operated by a remote switch such as a mobile phone, tablet app, or Bluetooth-enabled button. When the Bluetooth-enabled button is functioning, the two-way valve (D) is opened, and air containing the target odor is pushed from the vial (E) to join the ambient air and moves through the tubing (A) and out the odor port (B) via the pneumatic push connector (C). When the Bluetooth-enabled button is next pressed, the two-way valve (D) is closed, and air containing the target odor is stopped. Clean ambient air is pushed through the tubing (A) and out the odor port (B) via the pneumatic push connector (C) to clear the system.

The odor port (B) is attached to the front of a boat for the field investigation. The tube (A) leading from the port is run along the side of the boat, and at a convenient point over the side and into the boat to a box (O) that protects the device from water and being knocked.

The air intake tube from the air pump is fed through the box and outside the boat to sample ambient air from the local environment. When the power switch (K) is operated, the system starts to pump ambient air via the pump (G) through the system. The system also boots up the Arduino (N) and activates the Bluetooth connection so that the device can connect to the operating app. The system utilizes an “Arduino Manager” (16) application on an iPhone or iPad. Arduino Manager connects to the device via Bluetooth, allowing the odor valve to open and close by pressing an in-app button. The app supports simple coding and custom development of widgets. The code sketch running on the Arduino board allows the Arduino Manager app to send or receive data from the board and control the device.

Because the two-way electronic valve (D) is closed on startup, only ambient air is emitted via the odor port on the front of the boat. The ambient air is constantly supplied once the power switch is operated.

The sample glass Mason jar (E) contains the target material upon which the canine is trained (Figure 4). When the pneumatic push connector (D) is opened by the Bluetooth-enabled power control board (N), air from the air pump (G) is pushed into the jar; this forces the odor headspace of the target material out of the jar through the tube via Check Valve (J) and to the odor port (B). The sample vial can be any size of container to suit the requirements of the target training sample.

When the Arduino Manager in-app button is set to “on,” the two-way electronic valve opens and ambient air flows into the Mason jar containing the target odor. When the in-app button is set to off, the two-way electronic valve closes, preventing air from flowing into the Mason jar containing the target odor, meaning only ambient air is being pushed through the system. Once the air containing the target VOCs exits the port (B), it disseminates into the ambient air.

The flow of ambient air cleans the Teflon piping of any residual target odors and allows the system to be operated again (16). The cleaning process is timed at a minimum of 45 s to ensure the system contains no residual target odor. However, the flow rate can be calculated using the Continuity Equation for the steady-state flow equation, and for this device setup, it is calculated as 0.7 s, but factors such as the type of target odor used would have an influence on that time. Oil being very persistent resulted in extending the time to ensure there is no residual odor.”

In the published article, the in-text citation for reference (15) was not hyperlinked when first cited in **Section 2.1** Parts, paragraph 2. Also, the incorrect reference number 16 was used. The citation has now been corrected, and should read:

“The device is switched on to provide power to the Arduino Nano 33 IoT Bluetoothenabled on a custom printed Power Control Board (15) that serves as the operating system.”

In the published article, reference (15) was incorrectly cited in **Section 2.1** Parts, paragraph 9. The citation has now been corrected, and should read:

The system utilizes an “Arduino Manager” (16) application on an iPhone or iPad. Arduino Manager connects to the device via Bluetooth, allowing the odor valve to open and close by pressing an in-app button.

The original version of this article has been updated.

